# Association between body weight and distal gut microbes in Hainan black goats at weaning age

**DOI:** 10.3389/fmicb.2022.951473

**Published:** 2022-09-16

**Authors:** Lianbin Li, Kunpeng Li, Zhengyu Bian, Zeshi Chen, Boling Li, Ke Cui, Fengyang Wang

**Affiliations:** ^1^Key Laboratory of Tropical Animal Breeding and Epidemic Disease Research of Hainan Province, College of Animal Science and Technology, Hainan University, Haikou, Hainan, China; ^2^Hainan Extension Station of Animal Husbandry Technology, Haikou, Hainan, China

**Keywords:** gut microbiota, 16S rRNA, Hainan black goats, body weight, association

## Abstract

Gut microbiota plays a critical role in the healthy growth and development of young animals. However, there are few studies on the gut microbiota of young Hainan black goats. In this study, 12 three-month-old weaned lambs with the same birth date were selected and divided into the high body weight group (HW) and low body weight group (LW). The microbial diversity, composition, and predicted function in the feces of HW and LW groups were analyzed by collecting fecal samples and sequencing the 16S rRNA V3-V4 region. The results indicated that the HW group exhibited higher community diversity compared with the LW group, based on the Shannon index. The core phyla of the HW and LW groups were both *Firmicutes* and *Bacteroidetes*. *Parabacteroides*, *UCG-005*, and *Bacteroides* are the core genera of the HW group, and *Bacteroides*, *Escherichia-Shigella*, and *Akkermansia* are the core genera of the LW group. In addition, genera such as *Ruminococcus* and *Anaerotruncus*, which were positively correlated with body weight, were enriched in the HW group; those genera, such as *Akkermansia* and *Christensenellaceae*, which were negatively correlated with body weight, were enriched in the LW group. Differential analysis of the KEGG pathway showed that Amino Acid Metabolism, Energy Metabolism, Carbohydrate Metabolism, and Nucleotide Metabolism were enriched in the HW group, while Cellular Processes and Signaling, Lipid Metabolism, and Glycan Biosynthesis and Metabolism were enriched in the LW group. The results of this study revealed the gut microbial characteristics of Hainan black goats with different body weights at weaning age and identified the dominant flora that contributed to their growth.

## Introduction

The gut microbiota (GM) is the largest and most intricate micro-ecosystem in animals, of which bacteria are the most numerous (Bhattarai and Janaswamy, 2022). GM is considered to be the “hidden metabolic organ” of the body, which plays a significant role in physiological activities such as energy metabolism, nutrient digestion and absorption, immune regulation, and health maintenance (Bohan et al., 2019). GM can affect animal growth performance by regulating these physiological activities, and animal growth performance is an important factor affecting the profitability of animal husbandry.

Average daily gain (ADG) is an important indicator of animal growth in animal husbandry. Recently, one study found that GM is closely associated with ADG in animals (Fang et al., 2020). It has also been reported that some specific GMs can improve feed conversion rates and promote animal growth (Stanley et al., 2012). A study on swine found that early-life establishment of the gut microbiota can influence the phenotype of animals (Mach et al., 2015). Lu et al. (2018) studied the change trends of fecal microorganisms in weaned piglets, 15-week-old, and 18-week-old pigs by 16S sequencing and proved that GM is involved in the regulation of host body weight. Previous research has demonstrated that microorganisms may affect animal growth performance by regulating energy production in animals. For example, microorganisms can hydrolyze carbohydrates under anaerobic conditions and ferment them to generate short-chain fatty acids (SCFAs) (acetic acid, propionic acid, butyric acid), to provide energy for the host (Wang M. et al., 2019). Taken together, the regulatory effects of these GMs are all associated with the increase in animal body weight.

At present, there are few studies on the relationship between GM and body weight in sheep and goats. A recent study on sheep found that there were significant differences in the distribution and composition of intestinal flora in sheep with different body mass indices (Cheng et al., 2022). However, little research has been done on lambs. As we all know, young ruminants are in the period of the fastest growth of the body, the rapid improvement of the tissue and organ functions, and the accelerated construction of the immune system. Research has shown that early microbial exposure is a potentially effective intervention strategy to modulate host health and metabolism (Li et al., 2018). Manipulation of the early microbiome has been reported in newborn calves to improve growth performance and health (Malmuthuge et al., 2015). Therefore, the development of GM in young ruminants largely determines the product quality in adult animals.

Due to the huge quantity and complex composition of GM, it was impossible to accurately describe the composition and function of microorganisms by traditional methods in the past. Over the past ten years, the rise of 16S sequencing technology has greatly enriched the research content of GM. Many studies by 16S sequencing technology have analyzed the correlation between body weight and GM of weaned piglets and investigated the relationship between obesity and the GM of diabetic patients (Han et al., 2017; Pai et al., 2022). Therefore, 16S sequencing has become a well-established technique to study GM.

Hainan black goat is the dominant goat breed in Hainan Province, which has excellent qualities such as rough feeding resistance, strong disease resistance, and good meat performance. It is a valuable breed resource for large-scale breeding in tropical regions (Quanwei et al., 2019). However, Hainan black goat has the disadvantage of a slow growth rate, which becomes an important factor in restricting its industrial development.

Hence, we use 16S sequencing techniques for the sake of studying the traits of GM of Hainan Black goats with high body weight and low body weight. This study provides a theoretical basis for further determining the dominant flora and improving the growth traits of Hainan black goats at weaning age.

## Materials and methods

### Ethics statement

The sampling method and all subsequent methods were approved by the Ethical Committee of the Hainan University (Haikou, China, Permit number: HNUAUCC-2022-000122). This experiment did not involve any endangered or protected species.

### Experimental animals and sample collection

A total of 20 male Hainan black goats with the same birth date raised on a commercial farm in Hainan Province were used for this study. These lambs were self-bred by the farm with similar genetic backgrounds and fed with the ewes in each stage and suckled freely, after 21 days of age, they began to feed freely with the ewes on concentrate and high-quality forage and drank freely. Lambs were weighed when they reached 90 days of age. The fecal samples were collected using sterile tools and delivered to the lab within 2 h and stored at −80°C. All individuals were sorted according to body weight, and the six heaviest lambs were selected as the HW group (each sample was represented by H.1, H.2, H.3, H.4, H.5, H.6, respectively), and the six lightest ones as the LW group (each sample was represented by L.1, L.2, L.3, L.4, L.5, L.6, respectively). The average body weight of the HW group was 11.89 ± 1.67 Kg, and that in the LW group was 8.69 ± 1.22 Kg (Table 1). There was a significant difference in body weight (BW) and average daily gain (ADG) between HW and LW groups (*P* < 0.01; Supplementary Figure 1). No significant differences in the weight of the newborn lambs were observed. In the process of the experiment, the lambs were not exposed to any specific pathogens, diseases, or antibiotics.

**TABLE 1:** Body weights of the Hainan black goats used in this study.

Groups	Number	Body weight	Maximum	Minimum	*P* value
HW	6	11.89 ± 1.67	14.15	10.45	<0.01
LW	6	8.69 ± 1.22	9.95	7.15	<0.01

### DNA extraction

Genomic DNA was extracted from each sample by using TIANGEN^®^ Magnetic Soil and Stool DNA Kit (Qiagen, Valencia, California, USA). The quality of the extracted DNA was measured by 1% agarose gel electrophoresis.

### 16S rRNA gene polymerase chain reaction amplification, purification, and sequencing

In this study, the V3-V4 region was amplified by polymerase chain reaction (PCR), and the universal primer used was 341F:5′-CCTAYGGGRBGCASCAG-3′ and 806R:5′-GGACTACNNGGGTATCTAAT-3′. The total volume of the reaction was 30 μL, containing 15 μL Phusion Master Mix (2 ×), 3 μL Primer (2 μM), 10 μL DNA (1 ng/μL), and 2 μL H_2_O. After denaturation at 98°C for 1 min, 30 amplification cycles were performed comprising a denaturation step at 98°C for 10 s, an annealing step at 50°C for 30 s, an extension at 72°C for 30 s, followed by the last extension at 72°C for 5 min. 2% agarose gel electrophoresis was used to examine the quality of PCR products, and qualified PCR products were purified and quantified by enzyme labeling. The purified PCR products were then used to construct the sequencing library by TruSeq DNA PCR-Free Library Preparation Kit. Illumina NovaSeq6000 was used for sequencing after the library was qualified.

### Bioinformatics and data analysis

After splitting each sample data from the primer reads according to the Barcode sequence and PCR amplification primer sequence, truncating Barcode and primer sequence, using FLASH to splice, filter, and detect the reads of each sample, and finally, the chimera was eliminated to obtain the effective tags. Uparse algorithm was used to cluster all Effective Tags of all samples, and the sequence was clustered into OTUs with 97% consistency by default. Representative sequences for each OTU were taxonomically identified and phylogenetically analyzed. The alpha diversity indices [Chao1, Abundance-based coverage estimator (ACE), and Shannon index] of each sample were calculated using QIIME (Version 1.9.1), and the dilution curve and Rank abundance curve were drawn using R software (Version 2.15.3). Beta diversity was calculated using QIIME (Version 1.9.1) to compare the difference and similarities among different samples. Linear discriminant analysis effect size (LEfSe) was performed on the samples according to different grouping conditions. Based on the OTUs clustered from 16S rRNA sequencing data, this study employed a phylogenetic investigation of the community by reconstructing the unobserved state (PICRUSt) to infer the functional potential of the gut microbiota community. The inferred genes and their functions were precomputed in the database of the Kyoto Encyclopedia of Genes and Genomes (KEGG). *P* < 0.05 were considered to be statistically significant. GraphPad Prism (version 8.0c) and SPSS 17.0 were applied to statistical analysis. The data were expressed as means ± standard deviation (SD). The raw data of this study has been uploaded to the NCBI database under the number PRJNA827021.

## Results

### DNA sequences analysis

**FIGURE 1:**
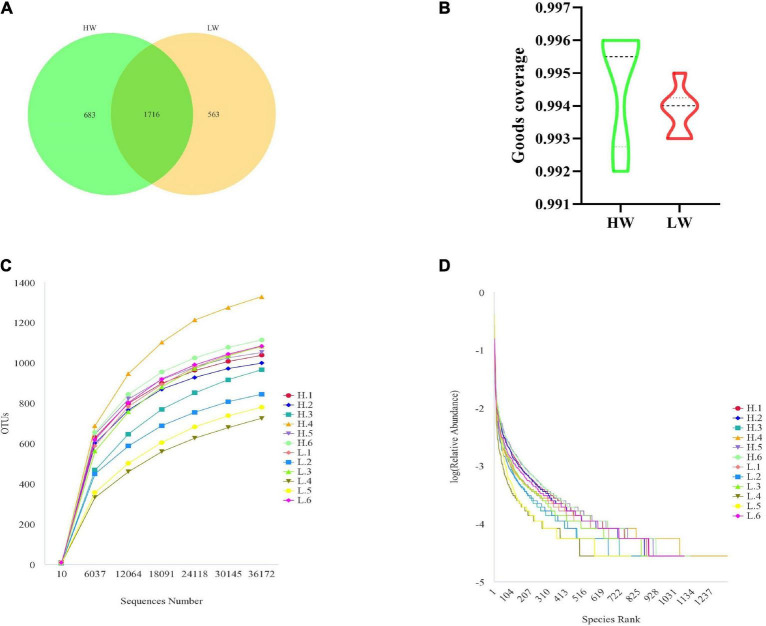
DNA sequence data analysis. **(A)** Venn diagram. The numbers in the figure show the unique or shared OTUs of each group. **(B)** It shows the sequencing depth of the HW and LW groups. Rarefaction **(C)** and rank-abundance curve **(D)** analysis of the different fecal samples at 97% sequences identity.

In this study, the 16S rRNA genes isolated from 12 fecal samples of Hainan black goats were sequenced and yielded 797799 sequences. After chimera checking and filtering out, 600862 sequences were produced from 12 samples and each sample had 50071 sequences on average. Following taxonomic assignment, the whole number of OTUs reached 2962 based on 97% nucleotide-sequence similarity and the HW and LW groups obtained 2399 and 2279 OTUs, respectively (Figure 1A). The OTUs of each sample in the HW and LW groups were shown in Supplementary Figure 2. The Good’s Coverage index values of all samples in groups HW and LW exceeded 99% (Figure 1B). The rarefaction curve presented a steady trend when the number of effective sequences overtops 36000. It indicated that the sequencing depth and quantity meet the requirement of the analysis (Figure 1C). Moreover, the rank abundance curve of each group of samples spanned a wide range in the horizontal direction and was relatively flat in the vertical direction, which indicated that the samples had good species richness and evenness (Figure 1D). These results indicated that the amount of sequencing data from this study was sufficient.

### Microbial diversity index analysis

**FIGURE 2:**
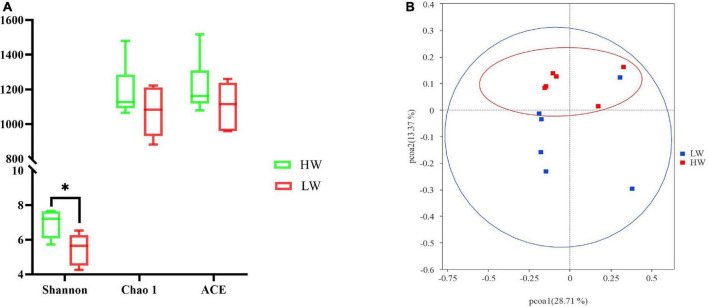
The different gut microbiota communities between the HW and LW groups. **(A)** Bacterial alpha diversity is based on Shannon diversity, Chao1, and ACE index. *Means the significant difference in statistics (*P* < 0.05). Shannon diversity represents microbial community diversity, and Chao1 and ACE represent species richness. **(B)** Differences in Principal Coordinate Analysis (PCoA) of GM structures HW and LW groups. The red dots represent the samples of the HW group and the blue dots represent the samples of the LW group. The distance between the two points represents the difference in GM.

For alpha diversity measurements of the GM population, the species abundance and community diversity were assessed by using the Chao1, ACE, and Shannon indices. We observed that the average of the Shannon index of group HW was 6.94 ± 0.80 and group LW was 5.48 ± 0.91 (Table 2). The Shannon index of HW was significantly higher than that of the LW group. Chao1 and ACE in group HW were 1065.74 ∼ 1479.21 and 1078.98 ∼ 1516.64, respectively, and those in group LW were 881.13 ∼ 1222.16 and 958.66 ∼ 1260.38, respectively. Although the Chao1 and ACE indices of HW were slightly higher than the LW group, the differences were not significant (Figure 2A).

**TABLE 2:** The alpha diversity index of each sample.

Sample	Shannon	Chao 1	ACE	Good’s coverage
H.1	7.25	1129.17	1131.68	0.996
H.2	7.16	1065.74	1078.96	0.996
H.3	5.72	1099.82	1186.89	0.993
H.4	6.21	1479.21	1516.64	0.992
H.5	7.66	1124.00	1137.05	0.996
H.6	7.65	1219.77	1240.85	0.995
L.1	5.96	1185.51	1229.84	0.994
L.2	5.36	947.86	1007.77	0.995
L.3	6.17	1222.16	1260.38	0.993
L.4	4.25	981.07	961.45	0.993
L.5	4.59	881.13	958.66	0.994
L.6	6.54	1206.28	1220.93	0.994

Combining the OTU species of the samples and their relative abundance, the PCoA was plotted based on the unweighted Unifrac distances. Figure 2B clearly showed the differences between all samples. The result showed that the HW group was mainly clustered on the upper side of the coordinate axis, while the LW group was mainly clustered on the lower side of the coordinate axis. The samples of the HW group and the LW group showed a relatively obvious separation. It indicated that the structure of GM was different between HW and LW groups.

### The analysis of community composition between high body weight group and low body weight group groups

**FIGURE 3:**
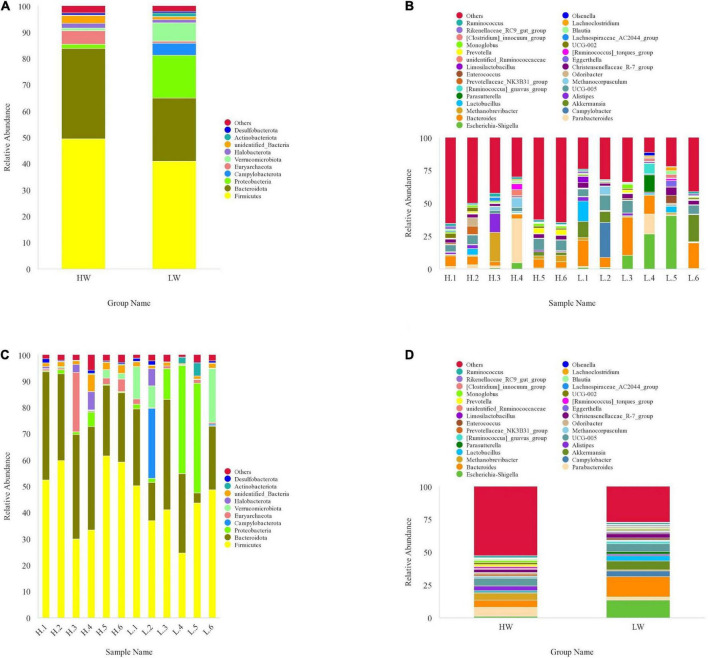
The taxonomic distribution between HW group and LW group samples (each color represents the relative abundance of a taxonomic bacterium). **(A)** at phylum level (top 10). **(B)** at genus level (top 30). **(C)** Between-group at phylum level (top 10). **(D)** Between-group at genus level (top 30).

The relative proportions of dominant taxa at phylum and genus levels were assessed by the distribution of microbial taxa in different groups. At the phylum level, we found 48 phyla in total in all samples from the HW and LW groups. According to the phylum assignment result (Figure 3), *Firmicutes* were the most abundant phylum of all samples, and *Bacteroidetes* were the secondary phylum. In the HW group, the relative abundance of *Firmicutes* (49.43%) and *Bacteroidetes* (34.42%) reached 83.85%, and the relative abundance of *Euryarchaeota* (5.23%) was also high. *Firmicutes* (40.93%) and *Bacteroidetes* (24.01%) remained in the highest relative abundance in the LW group, and interestingly, *Proteobacteria* (16.34%) reached equally high proportions in the LW group.

At the genus level, the core genera of the HW group were *Parabacteroides* (6.78%), *UCG-005* (5.84%), *Bacteroides* (5.51%), *Methanobrevibacter* (5.22%), *Alistipes* (3.49%), and *Christensenellaceae-R-7-group* (2.39%). The genera with high relative abundance in LW group mainly included *Bacteroides* (15.15%), *Escherichia-shigella* (13.36%), *Akkermansia* (6.87%), *UCG-005* (5.77%), *Campylobacter* (4.53%), *Lactobacillus* (3.69%), and *Christensenellaceae-R-7-group* (3.21%).

**FIGURE 4:**
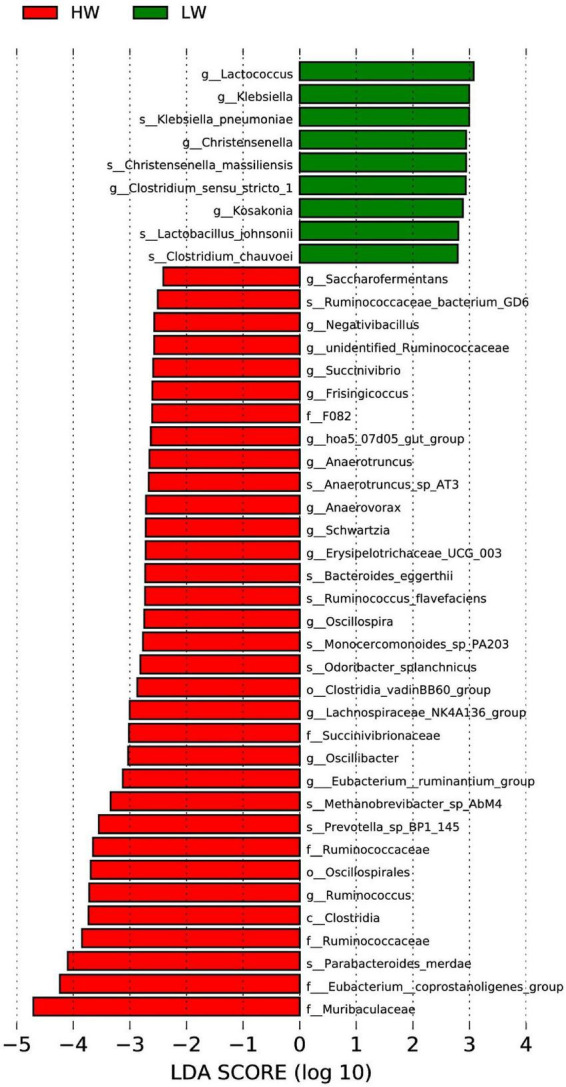
Significant differentiation of bacterial taxa between HW and LW groups was determined by linear discriminant analysis and effect size (LEfSe). LDA scores were calculated for bacterial taxa that were differentially enriched between different groups.

Differences between HW and LW groups were analyzed by Lefse analysis (LDA > 2) based on the classification of phylum and genus. Figure 4 showed that there was no significant difference between the HW and LW groups at the phylum and genus level; a total of 15 genera (e.g., *Ruminococcaceae*, *Oscillibacter*, *Oscillospia*, *unidentified-Ruminococcaceae*) were enriched in the HW group; 5 genera (e.g., *Lactococcus*, *Klebsiella*, *Christensenlla*) were enriched in the LW group.

### Spearman analysis

**FIGURE 5:**
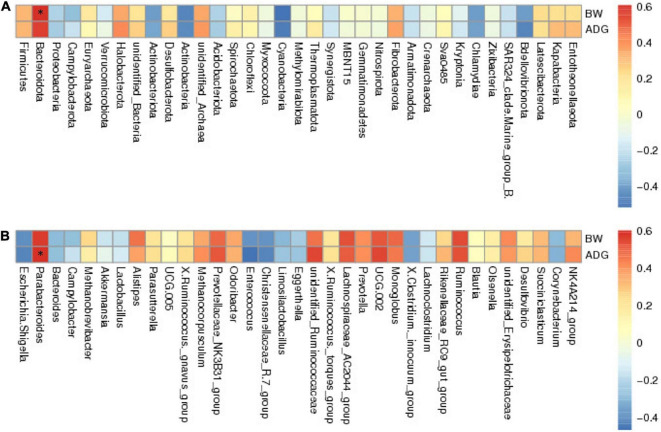
The results of Spearman’s analysis show BW (body weight) and ADG (average daily gain) in the longitudinal direction, and each bacterial information in the transverse direction. **(A)** at phylum level. **(B)** at genus level. The value corresponding to the intermediate heatmap is the spearman correlation coefficient r, which is between −1 and 1, *r* < 0 is negative correlation, *r* > 0 is positive correlation, and marked * indicates significance test *p* < 0.05.

To investigate the microflora that may be associated with growth performance, we performed Spearman’s rank correlation coefficient analysis between the GM and BW or ADG. The results showed *Firmicutes*, *Bacteroidetes*, and *Euryarchaeota* were positively correlated with BW and ADG; *Proteobacteria* was negatively correlated with BW and ADG at the phylum level (Figure 5A). At the genus level, we selected the top 35 genera correlated with BW and ADG, of which 23 genera had a positive correlation and 12 genera showed a negative correlation (Figure 5B). *Parabacteroides* showed the strongest positive correlation with BW and ADG. In addition, *Ruminococcus*, *Alistipes*, *UCG-005*, and *unidentified-Ruminococcaceae* were all positively correlated with BW and ADG. *Bacteroides*, *Akkermansia*, and *Lactobacillus* were negatively correlated with BW and ADG.

### Microbial function prediction

**FIGURE 6:**
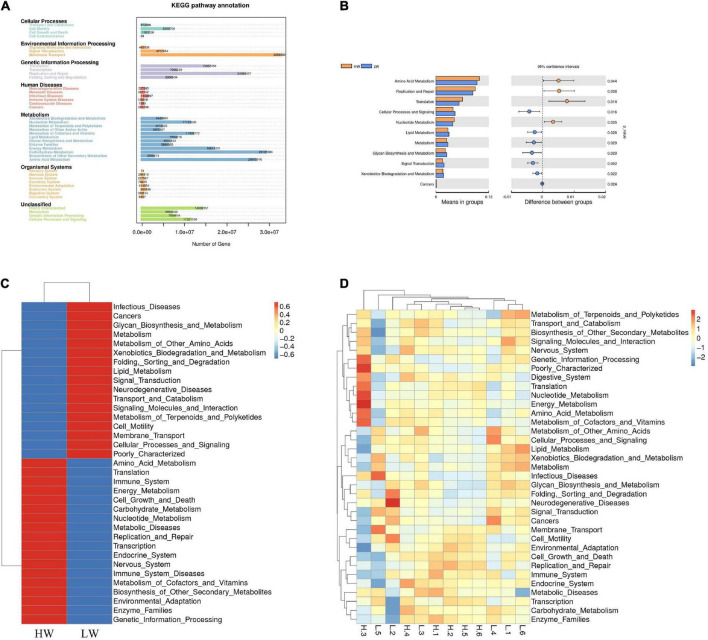
Genomic functional predictions. **(A)** KEGG pathway annotation. **(B)** Heatmap clustered based on functional prediction of each sample in level 2 KEGG pathway. **(C)** Heatmap clustered based on functional prediction of the different groups in level 2 pathway. **(D)** Comparison of HW and LW groups in differential metabolic pathways.

In this study, we used PICRUSt to predict and explore the molecular functions of microbial communities in two groups of samples. Figure 6A showed 41 functional genes predicted for Cellular Processes, Environmental Information Processing, Genetic Information Processing, Human Diseases, Metabolism, and Organismal Systems pathways. At the KEGG level 2, 41 functional genes in total were detected in the HW and LW groups. The majority of these functional genes belonged to Membrane Transport (11.78% of total genes inferred by PICRUSt), Carbohydrate Metabolism (10.54%), Amino Acid Metabolism (9.63%), Replication and Repair (8.71%), Energy Metabolism (6.81%), and Translation (5.73%).

The analysis showed that gut microbes in the HW group were mainly focused on Amino Acid Metabolism, Energy Metabolism, Carbohydrate Metabolism, Immune System, and Nucleotide Metabolism, while the gut microbes in the LW group were mainly focused on Infectious Diseases and Metabolism of Other Amino Acids (Figures 6B,C). We observed significant differences in many KEGG pathways in the fecal microbiota between the two groups by *T*-test (*P* < 0.05). The relative abundance of genes (Figure 6D) involved in Amino Acid Metabolism, Replication and Repair, Translation, and Nucleotide Metabolism in the HW group was significantly higher than in the LW group (*P* < 0.05), whereas the relative abundance of genes involved in Cellular Processes and Signaling, Lipid Metabolism and Glycan Biosynthesis and Metabolism were significantly lower than those in the LW group (*P* < 0.05).

## Discussion

Gut microbiota (GM) is known as the “second genome,” and its importance to the metabolic activities of animals is naturally self-evident (Zhu et al., 2010). Previous studies have shown that body weight changes in host animals can be affected by altering the gut microbes (Angelakis, 2017). The identification of GM that is beneficial for weight gain will provide an important aid to the healthy growth and production of animals. Therefore, in this study, 16S rRNA sequencing was used to analyze the composition of GM of Hainan black goats at weaning age and the differences in microbiota among goats of different body weights.

According to the results of the Shannon index, we found that the community diversity of the HW group was significantly higher than that of the LW group. Overall, the alpha diversity of the HW group was higher than that of the LW group, which was consistent with the research results on weaned piglets (Han et al., 2017). However, another study on rats found that the alpha diversity of obese rats was lower than that of lean rats (Resch et al., 2021). We speculate that this situation is due to possible differences in the composition and development of the GM between species. Furthermore, whether there is an inevitable correlation between GM alpha diversity and body weight needs further in-depth study.

At the phylum level, we found that *Firmicutes* and *Bacteroidetes* were dominant phyla in both the HW and LW groups, which was consistent with the results observed in Chinese small-tailed Han sheep, Mongolian sheep, and Boer goats (Zeng et al., 2015; Wang et al., 2018; Zhang et al., 2018). Although *Firmicutes* and *Bacteroidetes* were slightly more abundant in the HW group than in the LW group, the difference was not significant. Combined with the previous results, it was found that neither species nor body weight affected the dominant position of *Firmicutes* and *Bacteroidetes* in GM. *Firmicutes*, as the dominant bacteria in the intestine, were positively correlated with BW and ADG, which played a crucial role in the degradation and digestion of fiber and cellulose in animals (Hook et al., 2011). Another dominant phylum, *Bacteroidetes* had the main function of degrading non-fibrous carbohydrates and proteins and breaking down polysaccharides, thereby facilitating the digestion and absorption of nutrients (Huo et al., 2014). In addition, *Bacteroidetes* could also promote the formation of the intestinal mucosa and maintain the intestinal microecological balance of host animals (Béchon and Ghigo, 2022).

It is noteworthy that the relative abundance of *Euryarchaeota* was higher in the HW group, however, the relative abundance of *Proteobacteria* was higher in the LW group. By Spearman’s analysis, *Euryarchaeota* was positively correlated with body weight, and *Proteobacteria* was negatively correlated with body weight. Many studies found that *Euryarchaeota* accounts for a higher percentage of the obese group and it may be one of the strongest predictors of obesity measures, which is in agreement with our findings (Bortolin et al., 2018). *Proteobacteria* included many pathogenic bacteria, such as *Escherichia coli*, *Salmonella*, *Vibrio*, and *Helicobacter*, which were prone to cause metabolic disorders and inflammatory bowel disease (Rizzatti et al., 2017). Besides, *Proteobacteria* have been reported as a marker of microbial dysbiosis in GM (Shin et al., 2015). Therefore, we speculate that the high abundance of *Proteobacteria* might be a reason for the slow growth of lambs in the LW group.

By comparing the GM at the genus level, we found that most of the core genera were different in the HW and LW groups. *Parabacteroides* was the most abundant genus in the HW group. Spearman’s analysis showed that *Parabacteroides* had the strongest positive correlation with BW and ADG, suggesting that it was crucial to the improvement of lamb growth performance. Studies have found that *Parabacteroides* is a comprehensive probiotic that can produce succinic acid, generate propionic acid through hydrogenation, and has a positive regulatory effect on the glycolipid metabolism and protein synthesis (Wang M. et al., 2019).

*Ruminococcus* was significantly higher in the HW group than in the LW group, and it was positively correlated with BW and ADG. One study found higher *Ruminococcus* concentrations in obese patients, by comparing the fecal microbiota of the obese group and the control group in humans (Kasai et al., 2015). This was consistent with the results of this study. *Stanley et al.* found that *Ruminococcus* was enriched in the group with a high feed conversion ratio, indicating that *Ruminococcus* could indeed improve the digestion and absorption of feed and promote the increase of animal body weight (Stanley et al., 2016). Additionally, *Ruminococcus* was able to degrade indigestible fibers and fermented complex sugars by producing cellulases and played an important role in degrading resistant starch (Terry et al., 2019; Hong et al., 2022).

In addition, some genera enriched in the HW group were considered potential probiotics. For example, *Schwartzia* could ferment succinic acid to produce propionic acid, which provided energy for protein synthesis (Ran et al., 2021). *Anaerotruncus*, a succinate-producing bacterium, had a positive correlation with obesity (Goodrich et al., 2014; Shang et al., 2016). Some genera that were enriched in the LW group were also correlated with body weight. Studies have manifested that *Christensenellaceae* was enriched in individuals with low body mass index and was inversely correlated with obesity. We observed that *Akkermansia* was negatively correlated with BW and ADG. Previous studies have confirmed that *Akkermansia* can reduce the risk of diabetes, obesity, and inflammation by reducing the fat formation and inhibiting insulin, which may be a reason for the lower weight of lambs in the LW group (Derrien et al., 2017). Besides, there are many other genera enriched in the HW group, however, the functions of these genera are still unclear, and further research is needed.

The involvement of GM in different metabolic pathways may be a reason for the difference in lamb body weight. Therefore, we also analyzed the functional capacity of the GM. The results showed that at KEGG level 2, the HW group was enriched in Amino Acid Metabolism, Energy Metabolism, Carbohydrate Metabolism, and Nucleotide Metabolism, which was consistent with the findings in obese children (Li et al., 2021). Amino Acid Metabolism played a vital role in the synthesis of acetate, malonate, and butyrate, and could promote the production of SCFA in the intestine, thereby providing energy for the host (Neis et al., 2015). Meanwhile, Energy Metabolism and Carbohydrate Metabolism were beneficial to glycogen synthesis and glycolysis *in vivo* and ensured adequate energy intake and storage of animals (Cipolla-Neto et al., 2014; Mul et al., 2015). The metabolic pathways that were significantly enriched in the HW group were all essential routine metabolic functions of the body. At the same time, these metabolic pathways also proved that GM in the HW group had a more powerful capacity for food digestion and nutrient absorption than in the LW group.

It is well established that the differences in lamb body weight involved multiple factors, and GM is one of the core factors. Different GM may cause differences in lamb growth through host-microbe interactions, and PICRUSt provides a significant basis for identifying the function of goat gut bacterial communities. Notably, a large number of the bacteria detected belong to unclassified genera, therefore, weight-related genera still need to be continuously explored and studied.

## Conclusion

In this study, we compared the fecal microbiota of Hainan black goats in HW and LW groups at weaning age. There were significant differences in the microbiota structure between the two groups, and some microbes were detected to be significantly correlated with body weight. These results provide new information on the relationship between GM and growth traits of Hainan black goats at weaning age, and also provide directions for the use and development of gut microecological preparations.

## Data availability statement

The datasets presented in this study can be found in online repositories. The names of the repository/repositories and accession number(s) can be found below: https://www.ncbi.nlm.nih.gov/, PRJNA827021.

## Ethics statement

This animal study was reviewed and approved by the Ethical Committee of the Hainan University (Haikou, China, Permit number: HNUAUCC-2022-000122).

## Author contributions

LL and FW designed the experiments. LL, KL, ZB, ZC, and FW performed the experiments. KL and KC did the data analysis. LL, FW, and KL wrote the manuscript. All authors read and approved the final manuscript.
