# Supplementary material for: Association between body weight and distal gut microbes in Hainan black goats at weaning age
Source: Front Microbiol. 2022 Sep 16;13:951473. doi: 10.3389/fmicb.2022.951473 (PMC9523243; doi:10.3389/fmicb.2022.951473)
Supplement: Supplementary file 1 [file Image_1.pdf]

Supplementary Material

1.1 Supplementary Figures

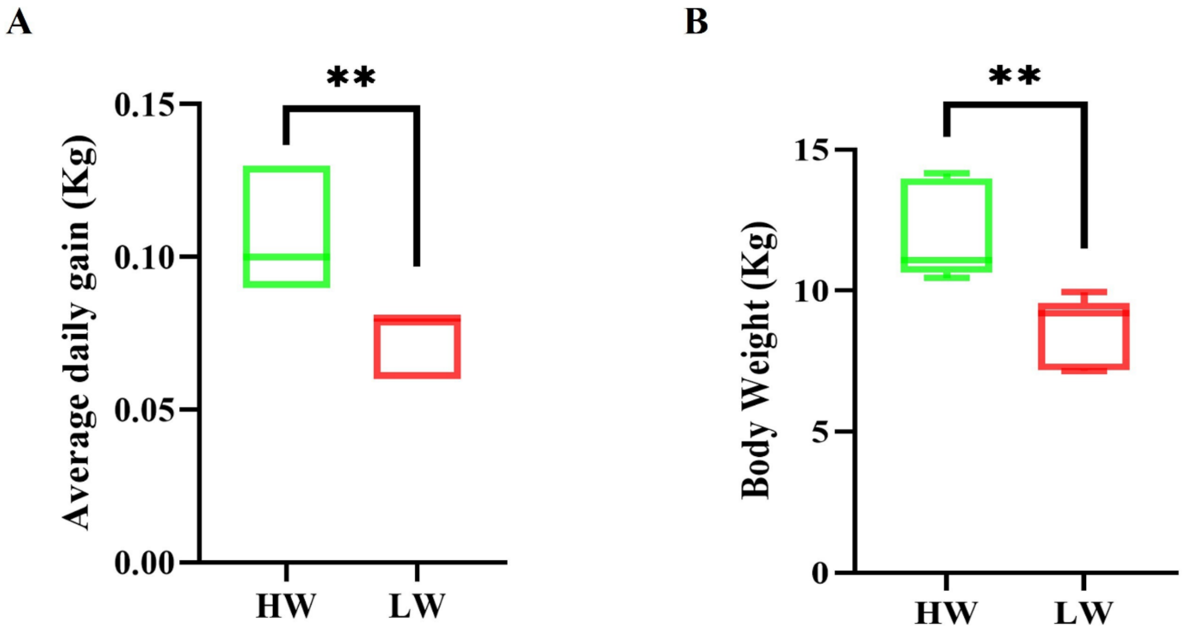

Figure S1. (A) Difference analysis of BW between HW group and LW group. (B) Difference analysis of ADG between HW group and LW group.

1.2 Supplementary Figures

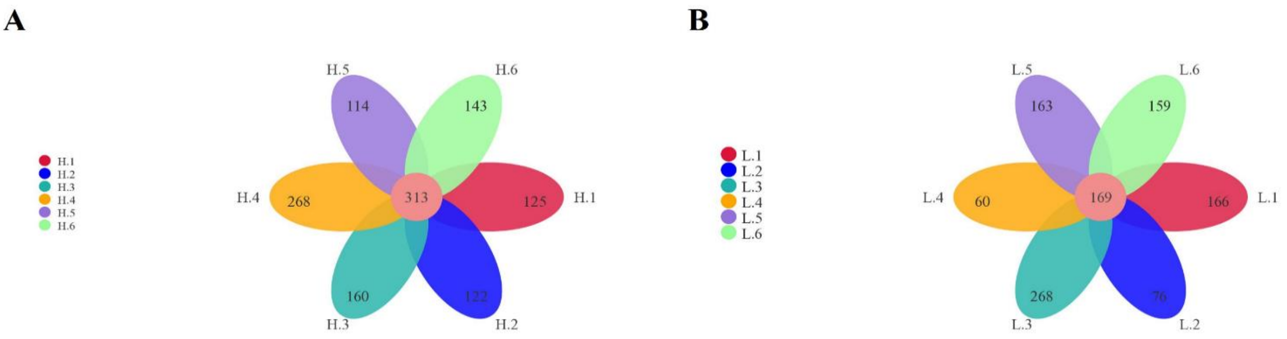

Figure S2. (A), (B) Venn diagram. The numbers in the figure show the unique or shared OTUs of each sample.
